# Expression profile of protein fractions in the developing kernel of normal, *Opaque-2* and quality protein maize

**DOI:** 10.1038/s41598-021-81906-0

**Published:** 2021-01-28

**Authors:** Mehak Sethi, Alla Singh, Harmanjot Kaur, Ramesh Kumar Phagna, Sujay Rakshit, Dharam Paul Chaudhary

**Affiliations:** 1 Department of Biochemistry, College of Basic Sciences and Humanities, Punjab Agricultural University, Ludhiana, 141004 Punjab India; 2 ICAR - Indian Institute of Maize Research, Ludhiana, 141004 Punjab India

**Keywords:** Biochemistry, Plant sciences

## Abstract

Maize protein quality is determined by the composition of its endosperm proteins, which are classified as nutritionally poor zeins (prolamin and prolamin-like) and nutritionally rich non-zeins (albumin, globulin, glutelin-like, and glutelin). Protein quality is considerably higher in *opaque-2* mutants due to increased content of non-zeins over zeins. However, the *opaque-2* endosperm is soft, which leads to poor agronomic performance and post-harvest infestation. Endosperm modification of *opaque-2* had led to the development of Quality Protein Maize (QPM), which has higher protein quality along with hard kernel endosperm. The present study was planned to analyze the expression dynamics of different protein fractions in the endospem of developing maize kernel in normal, *opaque-2* and QPM in response to the introgression of endosperm modifiers. Results revealed that albumin and globulin content decreases, whereas, prolamin, prolamin-like, glutelin-like, and glutelin content increases with kernel maturity. It has been observed that *opaque-2* mutation affects protein expression at initial stages, whereas, the effect of endosperm modifiers was observed at the intermediate and later stages of kernel development. It has also been noted that prolamin, glutelin, and glutelin-like fractions can be used as quick markers for quality assessment for differentiating QPM varieties, even at the immature stage of kernel development. Overall, the present study implicates the role of different protein fractions in developing and utilizing nutritionally improved maize varieties.

## Introduction

Maize (*Zea mays*) is known as the ‘queen of cereals’, which also has relevance as an industrial crop due to its highest grain yield potential^1^. It is a staple food for a large segment of the human population, especially in developing nations^2^. However, the quality of maize protein is poor as its biological value is just 40% of the milk protein casein^3^. As a result, protein-energy malnutrition is commonly observed in resource-poor nations where maize is consumed as a staple food^4^. A mature maize kernel constitutes 80–85% of endosperm, followed by germ (9–10%) and pericarp (5–6%). Endosperm, the largest part of maize kernel, is composed of 70% starch, 8–10% protein and is relatively low in fat content^5^. The germ also contains some protein whose composition is superior and better balanced as compared to that of an endosperm. However, its contribution towads overall protein quality is insignificant as the germ constitutes only 9–10% of the maize kernel. The endosperm lacks the essential amino acids, particularly lysine and tryptophan, due to the existence of higher proportion of nutritionally poor zeins than the nutritionally rich non-zeins^6^.

Maize endosperm consists of two types of protein i.e., zeins and non-zeins. Zein, the alcohol-soluble protein (prolamin, prolamin-like protein), is the major seed storage protein of maize kernel that constitutes approximately 50–70% of kernel endosperm, whereas, the non-zein protein consists of globulins (3%), glutelins (34%), and albumins (3%)^7, 8^. Generally, zein fraction contains higher proportion of leucine (18.7%), phenylalanine (5.2%), isoleucine (3.8%), valine (3.6%), and tyrosine (3.5%), but low amounts of essential amino acids such as threonine (3%), histidine, cysteine (1%), methionine (0.9%), and lysine (0.1%), and is almost devoid of tryptophan^9^. Lack of essential amino acids, particularly lysine and tryptophan and excess of leucine are responsible for the poor quality of zein proteins, whereas, non-zein fraction has a balanced proportion of essential amino acids^10^. Discovery of *opaque-2* mutant led to the development of nutritionally improved maize. *Opaque-2* maize had reduced zein protein (50–70%), specifically the 22-kDa alpha zeins, with a concomitant increase in non-zein fractions, including globulin, albumin and glutelins^11–13^. However, the pleiotropic effects associated with *opaque-2* mutants, such as soft and chalky endosperm, necessitated the development of Quality Protein Maize (QPM) with kernel texture similar to that of normal maize. At the same time, efforts have been made to ensure that protein quality in QPM remains near or at par with that of the *opaque*-2 maize^14, 15^. During maize kernel development, zein synthesis is reported to initiate at 12 days After Pollination (DAP), which gets stabilized towards maturity^16^. Zein proteins have a defined role in the formation of vitreous kernel texture by complexing to form starch-protein matrix^7^. The protein body formation initiates at 18–20 DAP with ordered recruitment of different zein fractions, where α- and δ-zeins occupy the central position, and γ- and β-zeins form the peripheral layer^17, 18^. Out of all zeins, the α-zeins (19- and 22-kDa) are the most abundant and are synthesized by four highly duplicated gene families, located across six chromosomes^19, 20^. Due to their high levels of expression and complexity, zein synthesis serves as a model system to analyze coordinated genetic regulation of several genes expressed at at a specific developmental stage^21^. Although a lot of information regarding the molecular mechanism of nutritionally improved maize is available^22–24^, differential expression of protein fractions under different genetic backgrounds with respect to endosperm development is not well understood. The expression profile of endosperm modifiers in QPM at different levels of kernel maturity was also not well understood in maize. The present study was designed to investigate the time course expression of protein fractions in the developing maize kernel of normal, *opaque-2* and QPM of Indian origin.

## Results and discussion

### Accumulation pattern of total protein and different fractions in developing maize kernel

**Figure 1:**
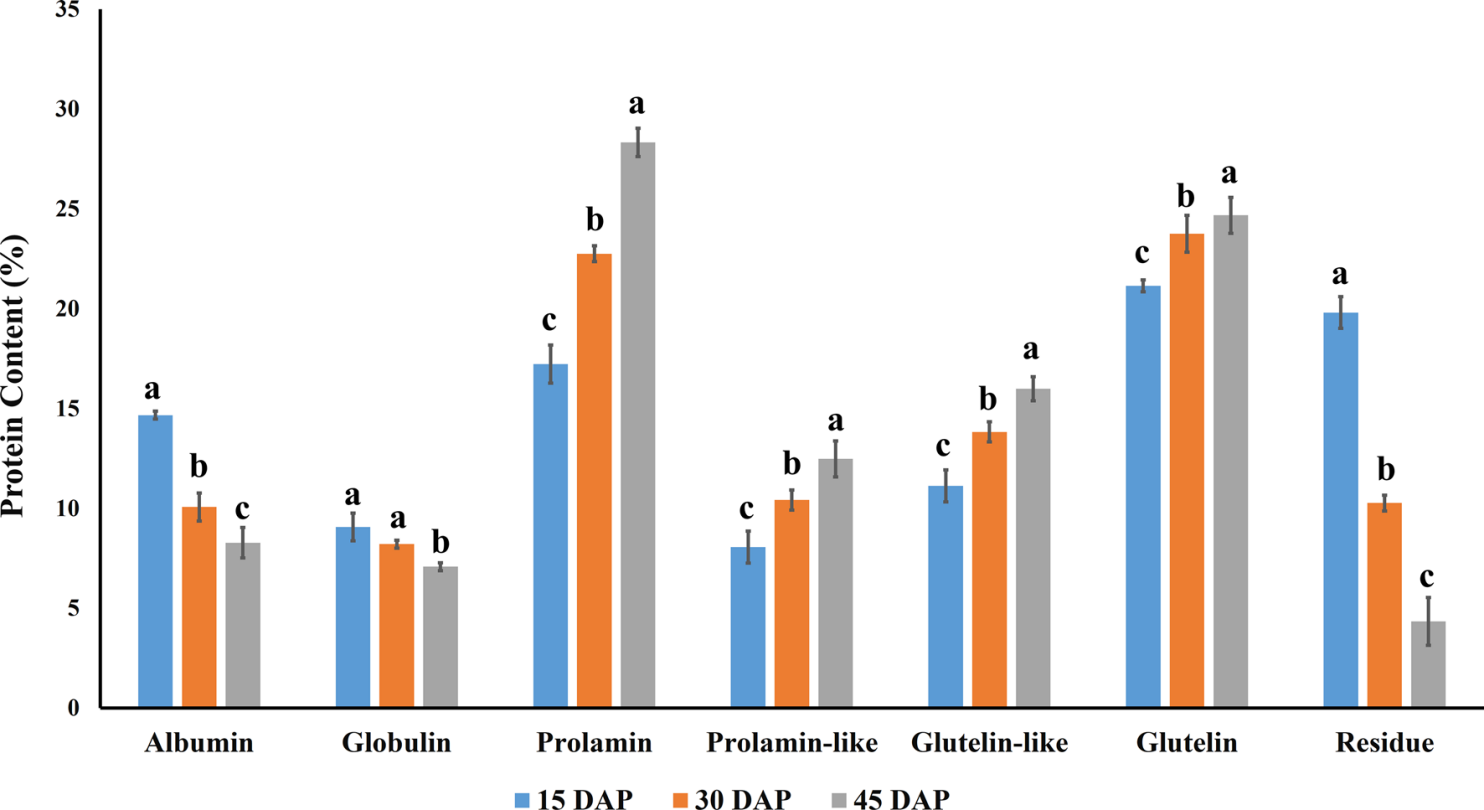
Trends of endosperm protein fractions accumulation at different stages of kernel development (Mean ± S.D.). Protein fractions are estimated by considering total protein percentage as 100% and total protein content is estimated on dry weight-basis. The small alphabets on top of the bar represent the significance level, as per critical difference. Identical alphabet represents non-significant variation at *P* < 0.05.

Protein concentration of different fractions (albumin, globulin, prolamin, prolamin-like, glutelin and glutelin-like) was calculated by assuming the total protein to be 100%. It was found that albumin and globulin proteins accumulate more during initial stages, then keeps decreasing with kernel maturity, whereas, accumulation of prolamin, prolamin-like, glutelin, and glutelin-like fractions were higher during the later stages of kernel development (Fig. 1). It was observed that albumin and globulin may have contained the metabolically active proteins including enzymes or signal proteins which are important at the initial actively dividing phase of kernel development as defined by their accumulation pattern, but the mature kernel showed less activity in terms of metabolism, so albumin and globulin degraded with kernel development. Since zein proteins (prolamin, prolamin-like proteins) are the major storage protein of maize kernel^25^, expectedly their synthesis increases with kernel development as reflected by our results. In confirmation to our findings, it has previously been reported that zein accumulation increases from 10 to 45 (DAP)^25, 26^. Further, glutelins also showed an increasing trend of accumulation with kernel development indicating that like zeins, they also serves as a storage protein and may act as an energy reservoir for mature seeds. Residue protein also decreases with kernel development, indicating that many uncharacterized proteins that may be involved during initial stages, starts declining as the kernel reaches the dormant stage of development (Supplementary Table S1).

### Comparison of Zein content in Normal, *opaque-2*, QPM lines in developing kernel

**Figure 2:**
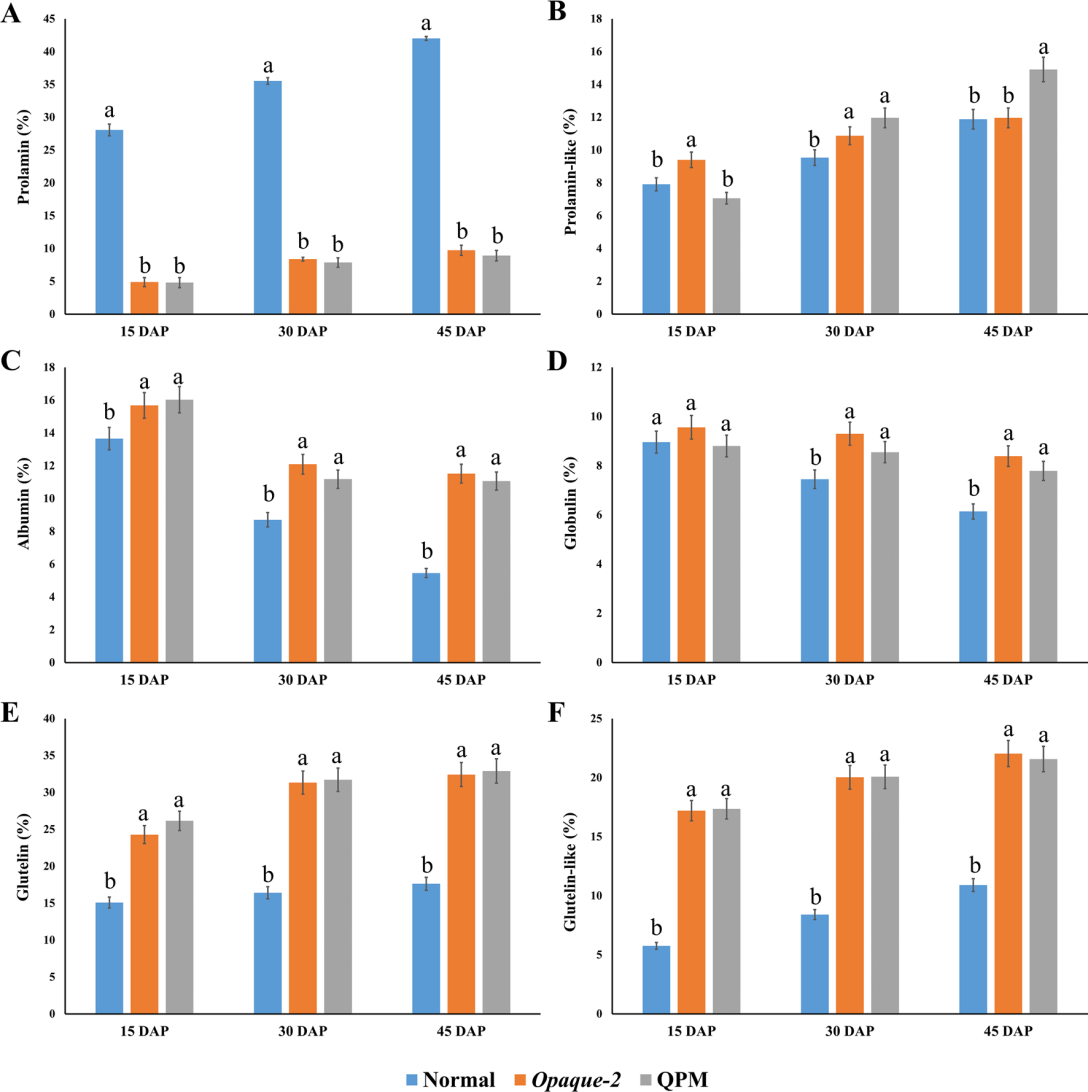
Comparison of protein fractions including zein (**A**, **B**) and non-zein (**C**, **D**, **E**, **F**) between normal, *opaque-2* and QPM lines at different stages of kernel development. Error bars denote ± SD of three replicates. The small alphabets on top of the bar represent the significance level, as per critical difference. Identical alphabet represents non-significant variation at *P* < 0.05.

Protein fractions are broadly classified into zein (nutritionally poor) and non-zein (nutritionally rich). The major endosperm storage protein, the prolamin and prolamin-like fractions, are collectively referred to as zeins. These proteins contain a high concentration of amino acids such as glutamine, proline, leucine, and alanine, but are deficient in essential amino acids and therefore they have relatively poor nutritional quality^9^. Our data clearly shows that prolamin fraction was maximally retained in normal as compared to *opaque-2* maize at each stage of kernel development (Fig. 2A) with fold differences of 5.74 at 15 DAP, 4.24 at 30 DAP, and 4.32 at 45 DAP. A similar trend of differential prolamin accumulation was observed between normal and QPM lines, with normal lines retaining more prolamin with fold difference of 5.84 at 15 DAP, 4.51 at 30 DAP, and 4.71 at 45 DAP. Although zeins (prolamins and prolamine-like) are nutritionally poor but they significantly contributes towards kernel hardness as reported by Guo et al. (2013) who showed a direct correlation between overall zein content and kernel vitreousness, demonstrating that normal maize with vitreous kernel texture retained more zein content as compared to *opaque-2* mutants^27^. Next, it was observed that *opaque-2* and QPM had equal amounts of prolamin at initial stages (i.e. at 15 DAP), but when the kernel matured, *opaque-2* retained slightly higher prolamin content as compared to QPM, with a fold change of 1.06 at 30 DAP and 1.09 at 45 DAP (Fig. 2A). Overall, the expression profile of prolamin shows that normal maize differs from *opaque-2* and QPM at each developmental stage, but the maximum difference was observed at the initial stages of kernel development.

Considering the fact that prolamin-like fraction includes sulfur-rich proteins, we next analyzed the prolamin content among *opaque-2* and QPM lines. The data depicted in Fig. 2B indicates that there was no uniform trend in accumulation pattern of prolamin as *opaque-2* retained more prolamin-like fraction than normal (1.18 fold difference) and QPM (1.32 fold difference) at 15 DAP, whereas, normal and QPM lines retained almost similar content of prolamin-like fraction at 15 DAP. At 30 DAP, QPM retained more prolamin-like fraction compared to normal (1.13 fold difference) and *opaque-2* (1.65 fold difference), whereas *opaque-2* retained more prolamin-like fraction than normal lines at 30 DAP with a 1.14 fold difference. At 45 DAP, prolamin-like content accumulated maximally in QPM lines in comparison to *opaque-2* and normal germplasm, with a 1.27 and 1.25 fold difference, respectively. Normal and *opaque-2* lines accumulate an almost similar quantity of prolamin-like fraction at maturity. It was observed that QPM accumulates a higher level of the prolamin-like fraction at 30 and 45 DAP, indicating that endosperm modifiers may start getting expressed at intermediate stages of kernel development. It has been reported that QPM lines have 2–threefold higher concentration of 27-kDa γ-zein (which falls under prolamin-like fraction) as compared to wild type and *opaque-2* mutants^28^. 27-kDa γ-zein is a member of cysteine-rich proteins and plays an important role in retaining the starch-protein matrix in QPM by forming disulphide bridges and has been reported to be correlated closely with vitreous kernel texture^29^. It has also been reported that one major QTL for endosperm modification was found to be associated with 27-kDa γ-zein, which has a role in vitreous kernel texture in QPM^30, 31^. It has also been observed that normal lines have a higher level of residue protein content as compared to *opaque-2* and QPM lines, which revealed that introgression of *opaque-2* mutant and endosperm modifiers reduces the residue protein content along with a reduction in zein fraction. (Supplementary Table S1).

### Effect of genetic background on zein accumulation among different maize types

The present study revealed that genetic background adds variability in terms of zein expression at 45 DAP as prolamin content varied from 40.17% in HKI 1105(N) to 45.22% (CML 117) within normal lines (1.12 fold difference) (Supplementary Table S1). Similarly, in *opaque-2* genotypes, it varied from 8.62 (CML 269) to 10.64 (HKI 323) with a 1.22 fold difference. In QPM, a variation of 8.15% (LM 11 263B) to 10.23% (HKI 1105 QPM) has been observed with a 1.26 fold difference. Prolamin-like fraction varied from 10.25% (LM 11 1275) to 13.96% (CML 117) in normal lines with a 1.36 fold difference, 10.27% (CML 269) to 12.95% (VQL 1) (1.25-fold difference) in *opaque-2* lines and from 13.15% (DQL 1019) to 18.44% (HKI 1105 QPM) in QPM genotypes with a 1.4 fold difference (Supplementary Table S1). For both prolamin and prolamin-like fractions, variability was maximally observed within QPM germplasm which proves that the endosperm modifiers play a major role in modulating the expression of zein synthesis. The present information can be implemented for targeting specific endosperm modifiers to enhance kernel vitreousness without compromising protein quality.

It is clearly evident from the data presented in Fig. 2B that the prolamin-like protein fraction accumulates rapidly towards the intermediate and later stages of kernel development in QPM when compared to *opaque-2*. This indicates its possible role in endosperm modification.

### Comparison of non-Zein content in normal, *opaque-2* and QPM lines in developing kernel

A comparison of albumin content among different maize types is represented in Fig. 2C. At 15 DAP, albumin content was observed maximally in QPM lines with 1.17 and 1.02 fold increase, as compared to normal and *opaque-2* lines, respectively. Higher albumin with fold difference of 1.15 was observed in *opaque-2*, when compared to normal lines. At 30 and 45 DAP, *opaque-2* and QPM retained higher albumin content in comparison to normal lines, whereas, *opaque-2* and QPM retained almost similar albumin content at these stages. From above, it is inferred that albumin degrades with kernel development in all maize types as shown in Fig. 1, but degradation is maximal in normal lines as compared to *opaque-2* and QPM counterparts. Globulin accumulation pattern was similar to that of albumin, as *opaque-2* and QPM retained higher globulin content than normal lines majorly towards kernel maturity (Fig. 2D).

Glutelin-like fraction increases with kernel development and appears to be an important protein fraction affected by *opaque-2* mutation. At 15 DAP, *opaque-2* retained higher glutelin-like protein as compared to normal lines with a 2.99 fold difference. Among QPM and normal lines, QPM retained more glutelin-like proteins with a 3.01 fold difference (Fig. 2F). In the maturing kernel, *opaque-2* retained more glutelin-like content as compared to normal lines, with a 2.37 fold increase at 30 DAP and a 2.01 fold increase at 45 DAP. QPM also followed a similar trend for the accumulation of this fraction with 2.39 fold change at 30 DAP and a 1.99 fold difference at 45 DAP as compared to normal lines. *Opaque-2* and QPM have similar glutelin-like content. Glutelin fraction also had a similar accumulation pattern as that of Glutelin-like content, with *opaque-2* and QPM having similar glutelin content and normal maize having less glutelin content at each stage of kernel development (Fig. 2E). Similar findings have been reported earlier indicating that glutelin content increased from 17 to 44% in *opaque-2* mutants as compared to its normal counterpart^32^.

From the above results, it is observed that *opaque-2* mutation majorly affected prolamin and glutelin fractions (Fig. 2). It has been reported that *opaque-2* mutation leads to an overall reduction of zein protein, specifically the prolamins (22-kDa α-zein) and the compensatory mechanism subsequently leads to non-zein synthesis, majorly glutelin. Thus, *opaque-2* and normal lines are majorly differentiated from each other in terms of prolamin and glutelin content^12, 13, 32^.

### Effect of genetic background on non-zein accumulation among different maize types

The effect of diverse genetic background on the accumulation of non-zeins at kernel maturity stage (45 DAP) revealed that the albumin content varied from 4.31% (HKI 323 N) to 6.87% (CML 172) (1.58 fold difference) among normal lines, from 10.24% (CML 269) to 12.93% (HKI 1128) (1.25 fold difference) in *opaque-2* and from 9.15% (LM12 177) to 14.25% (HKI 1105) (1.56-fold difference) in QPM lines. Within normal lines, globulin content ranges from 4.53% (HKI 323 N) to 6.96% (LM11 1275) with 1.57 fold difference, in *opaque-2* mutant from 8.04% (HKI 323) to 9.04% (HKI 1128) with 1.12 fold difference, whereas in QPM it ranges from 6.94% (HKI 1105) to 8.83% (VQL 2) with 1.26 fold difference (Supplementary Table S2). Maximum variability was observed within the normal background, whereas *opaque-2* and QPM do not exhibit much variability in the accumulation of albumin and globulin content, which indicates that albumin and globulin are least affected by *opaque-2* mutation and endosperm modifiers.

Glutelin-like content ranges from 9.95% (LM 12) to 12.43% (CML 334) (1.24 fold difference) in normal lines, from 20.34% (DQL 1005) to 23.51% (HKI 323) (1.5 fold difference) in *opaque-2* and from 19.93% (LM 11 236B) to 24.66% (VQL 2) (1.24 fold difference) in QPM genotypes. Genetic variation was again found to influence the glutelin content as significant variation has been observed among normal {11.12% (CML 266) to 19.65% (HKI 323 N) (1.81 fold difference)}, *opaque-2* {29.45% (HKI 1128) to 36.65% (CML 269) (1.24 fold difference)} and QPM lines {31.13% (VQL 2) to 34.43% (LM11 288) (1.11 fold difference)} (Supplementary Table S1). For glutelin and glutelin-like protein contents, maximum variability was observed within *opaque-2* germplasm as compared to normal and QPM counterparts. From the present results, it is inferred that *opaque-2* mutation leads to variable increases in glutelin fractions as a compensatory mechanism to cope up decrease in prolamin fractions.

### Protein marker for quality assurance and QPM development

**Figure 3:**
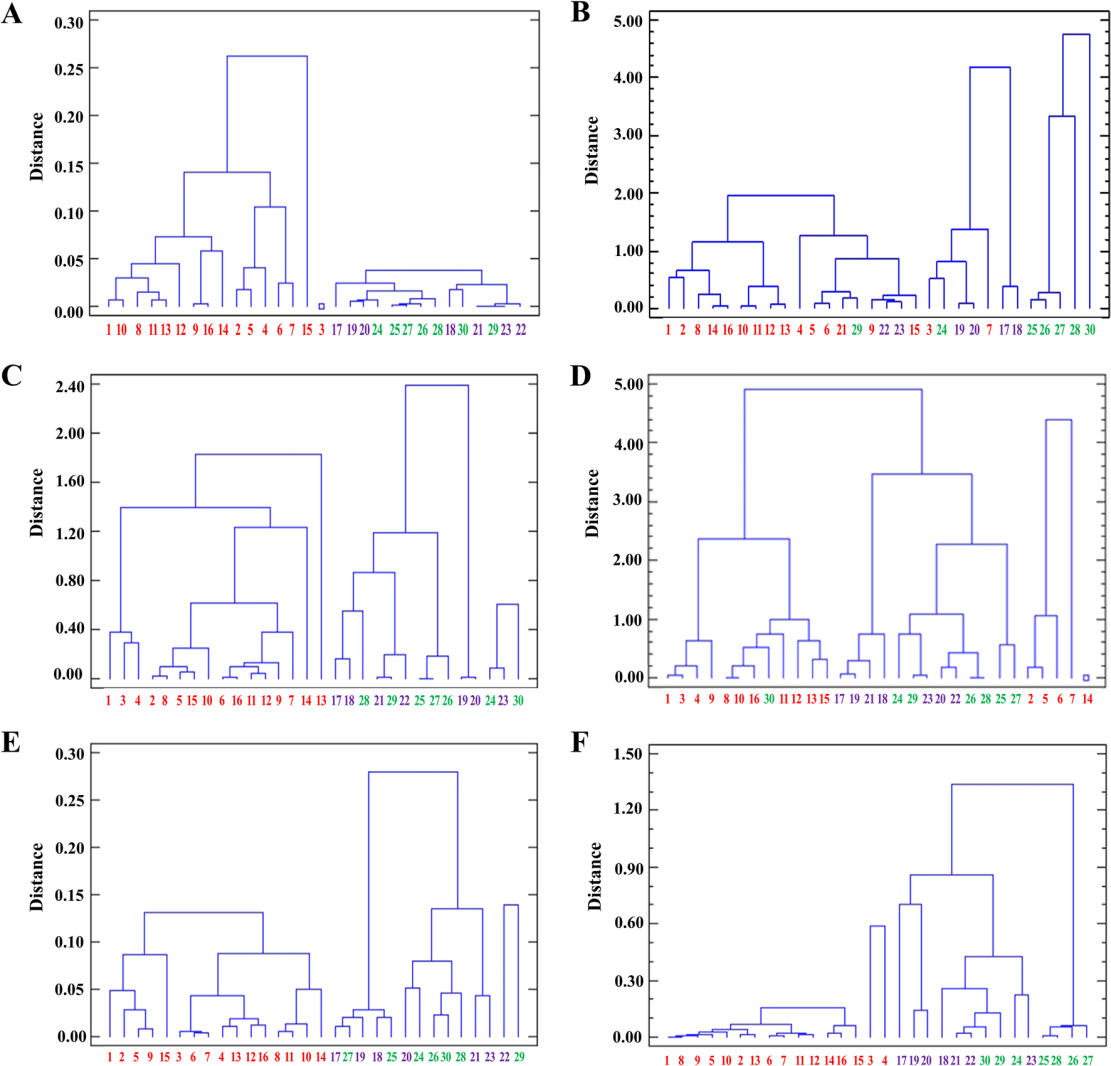
Dendrogram of normal (1–16), *opaque-2* (17–23), QPM (24–30) lines based on prolamin (**A**), prolamine-like (**B**), albumin (**C**), globulin (**D**), glutelin-like (**E**) and glutelin fractions (**F**). Dendrogram analysis on group average, squared euclidean basis. For numbering of normal, *opaque-2* and QPM genotypes, refer Supplementary Table 1.

Maize is increasingly being used for human consumption, so QPM has many potential health and nutrition benefits, and can be utilized as a commercial nutritious product. However, a mechanism is needed, whereby the normal maize can be differentiated from QPM, so that the industry can develop value chains for procurement and stocking of QPM. It is observed that among different protein fractions, albumin, prolamin, glutelin and glutelin-like fractions can differentiate normal maize from high protein-quality maize, i.e. *opaque-2* and QPM lines (Fig. 3A,C,E,F). In normal maize, the prolamin fraction is higher, while the albumin, glutelin and glutelin-like fractions are lower when compared to *opaque-2* and QPM. Figure 2A,C,E,F also revealed that while albumin fraction can be used to differentiate normal maize from *opaque-2* and QPM at maturity stage only, prolamin, glutelin and glutelin-like fractions are able to differentiate normal from high protein quality maize at all stages of kernel development, viz*.*, 15, 30 and 45 DAP. Hence, these fractions can be utilized by plant breeders and biochemists as a decision–making tools to assess the quality of standing crops, even at the early stages of kernel development. Based on the quality assessment at boot stage, the crop may be harvested for the kernel or in case the quality deteriorates due to pollen contamination the crop may be harvested for silage or stover. Figure 3B also suggests that prolamin-like protein fraction may be standardized for differentiation of all the three genotypes, viz*.*, normal, *opaque-2* and QPM. However, at early stages (15 DAP), it does not appear to differentiate well, limiting its use as a marker for protein quality (Fig. 2B).

The prolamin-like protein contains 27 KDa gamma zein, which is reported to be retained more in QPM as compared to normal and *opaque-2* lines^33^. Fast and precise segregation of QPM from *opaque-2* will be of immense use in expediting the QPM development program, as hard kernel QPM is a prerequisite for the development of high yielding, insect-resistant nutritionally improved maize. Dendrogram analysis also revealed that genetic background plays an important role in modifying the nutritional quality of maize. It is already reported that seven QTLs are associated with endosperm modification and expression of a particular modifier is affected by genetic background^28^. Overall, it shows that *opaque-2* mutation acts on broad-spectrum and its outcome is dependent on the genetic backgrounds. The converted QPM lines showed a variable trend in the accumulation of protein fractions, which proved that endosperm modifiers have a more complex mode of inheritance. Endosperm modifier genes are quantitative trait loci (QTL) which show additive effect and its influence varies with respect to biochemical composition of maize and genetic backgrounds^34^. QTLs for endosperm modification are variably expressed under different genetic backgrounds and deletion mutagenesis can be an additional way to confirm suspected *opaque-2* endosperm modifiers and their potential for endosperm modification under different genetic backgrounds^7^. Dendrogram for globulin fraction (Fig. 3D) represents that no separate clusters are formed for normal *opaque-2* and QPM lines. Therefore, globulin cannot be used as a protein marker for differentiation of different maize types.

The broad comparison of zein and non-zein fractions in normal, *opaque-2* and QPM lines at kernel maturity revealed that normal lines retained a higher concentration of zein and low concentration of non-zein proteins, whereas *opaque-2* and QPM showed a high concentration of non-zein and least concentration of zein proteins. It was reported that *opaque-2* mutation causes a decrease in zein synthesis and an increase in the accumulation of many other endosperm proteins (non-zeins) as zeins do not contain lysine, all the protein-bound lysine comes from the non-zein fraction^12, 35^. Hence, the reduction in zein and the increased synthesis of non-zein proteins both contribute to the enhanced percentage of lysine in the grain protein.

## Conclusion

From the above results, it is concluded that nutritionally poor zein fraction is a major storage protein found to be in highest concentration in normal maize, and least in *opaque-2*, whereas QPM showed intermediate content of zein towards kernel maturity. Nutritionally rich non-zein protein fraction is present maximally in the *opaque-2*, intermittently in QPM, and least in normal lines. The presence of high content of nutritionally poor zein decreases protein quality of normal lines as compared to *opaque-2* lines, where a reduction in zein content is compensated by an increase in the amount of non-zein proteins. The overall protein quality of maize kernel is found to be superior at the early stages of kernel development, as zein proteins are synthesized in the later stages of endosperm development. The milking stage kernel possesses both higher content of protein quantity and quality even in normal maize. A high concentration of prolamin-like protein fraction retained by QPM lines is supposed to play an important role in modifying the kernel texture of QPM at maturity. The fold difference observed among normal lines for prolamin content explains that although *opaque-2* transcription factor is considered to be a major player for prolamin accumulation, other accessory factors must work in collaboration with *opaque-2* to give differential prolamin expression under different genetic background in normal maize. These factors must be studied for the detailed elucidation of prolamin expression, which can be applied for the development of high yielding nutritionally rich maize varieties. We also found that the prolamin, glutelin and glutelin-like protein fractions can be used as precise biochemical markers to distinguish normal from *opaque-2* and QPM lines, whereas prolamin-like fraction can be established as a potent marker to distinguish all the three genotypes, viz*.*, normal, QPM and *opaque-2*. Overall, the dynamics of different protein fractions observed in the present study have implications for developing and monitoring the progress of high protein-quality maize. The use of nutritionally rich maize varieties for the synthesis of nutritious products having protein quality at par to milk protein casein will immensely benefit the end use consumers and other stakeholders.

## Materials and methods

### Experimental materials

Experimental material was procured from Indian Agricultural Research Institute, New Delhi, Indian Institute of Maize Research, Ludhiana and Punjab Agricultural University, Ludhiana. A total of thirty lines including 16 of normal maize, 7 *opaque-2* and 7 QPM with different genetic backgrounds were grown in the experimental fields of ICAR-Indian Institute of Maize Research, Ludhiana during *Kharif* 2017. The material was selected based upon its agronomic performance and better combining ability characteristics. The complete detail of the material including the opaqueness is presented in supplementary data. Normal maize harnesses a rigid matrix and as a result, showed 0% opaqueness, *opaque-2* mutants owing to distort starch-protein matrix showed 100% opaqueness, whereas QPM lines have a varying degree of opaqueness due to differential expression of endosperm modifiers^36^. The experimental material was grown in the field in a plot size of 21m^2^ with a row length of 3 m. Self-pollinated ears (minimum of 8) from each accession were collected at 15, 30 and 45 days after pollination (DAP), and frozen immediately in liquid nitrogen. Kernels were extracted from the center of each cob; the endosperm was dissected and freeze-dried using a lyophilizer (Freezone 2.5) and stored at − 20 °C. To minimize the effect of biological variation between ears, equal numbers of dissected endosperms from 4 ears of each line were pooled and treated as one sample; and a minimum of three replicated samples was taken for each experimental analysis. Extracted endosperms were finely ground and defatted using petroleum ether (40–60 °C).

### Protein fractionation and estimation

Protein fractionations were estimated as per the method of Landry and Mourex (1970)^37^ with certain modifications. Based on the solubility characteristics, maize endosperm proteins are broadly classified into five fractions including water-soluble (albumins), salt soluble (globulins), alcohol soluble (prolamin, prolamin-like), alkali-soluble (glutelins, glutelin-like), and residue (insoluble) proteins. For this purpose, 2 g of defatted finely ground sample was mixed into 10 ml distilled water and vortexed for 60 min at 4 °C. The supernatant was collected after centrifugation at 6000 rpm for 5 min. The extraction was repeated twice with the pellet obtained above and the supernatant thus obtained represents fraction I (albumin). Subsequent fractions were extracted from the residue of fraction I following the same procedure with different solvents: sodium chloride for globulins (fraction II), isopropanol 70% for prolamin (fraction III), isopropanol, 70% + 2-mercaptoethanol 0.6% (v/v) for prolamin- like (fraction IV), borate buffer 0.05 M (pH 10) + 2-mercaptoethanol 0.6% (v/v) for glutelin- like (fraction V) and borate buffer (pH 10), 2-mercaptoethanol, 0.65 (v/v), sodium dodecyl sulphate 0.5% (w/v) for glutelin (fraction VI). Along with these fractions, residue protein was also estimated. All the fractions were subsequently processed for nitrogen estimation to obtain protein content by micro-Kjeldahl method (AOAC 1975). The protein concentration of the above-mentioned fractions was estimated by considering the total protein percentage as 100% and the total protein content of each accession was reported earlier^24^.

### Statistical analysis

Repeated measure analysis of descriptive statistics and two-way analysis of variance (ANOVA) along with Tukey’s posthoc test among different experimental genotypes at different stages of kernel development was done using SPSS software. The data represented in Figs. 1 and 2 is reported at a 5% significant level and critical difference was calculated using CPCS-I software. The highest value among a group in Figs.1 and 2 has been assigned letter a, followed by b and c. Same letter within groups denotes absence of significant different at *P* < 0.05. Dendrogram analysis was performed using group average squared Euclidean method by using statgraphics18 software (Statistical Graphics Corp. Manugistics Inc., Cambridge, MA) to describe the genetic distance between thirty experimental lines for each protein fraction. Group clustering method was applied to make clusters, in which members within one cluster had similar expression pattern (Fig. 3). The average between members of one cluster was compared with the average between members of other clusters.

## Supplementary Information

Supplementary Information
