# Supplementary material for: Expression profile of protein fractions in the developing kernel of normal, Opaque-2 and quality protein maize
Source: Sci Rep. 2021 Jan 28;11:2469. doi: 10.1038/s41598-021-81906-0 (PMC7844038; doi:10.1038/s41598-021-81906-0)
Supplement: Supplementary file 1 — Supplementary Information [file 41598_2021_81906_MOESM1_ESM.docx]

**Expression Profile of Protein Fractions in the Developing Kernel of Normal, *Opaque-2* and Quality Protein Maize**

Mehak sethi^a^, Alla Singh^b^, Harmanjot Kaur^a^, Ramesh Kumar^b^, Sujay Rakshit^b^ and Dharam Paul Chaudhary^b^*

a Department of Biochemistry, College of Basic Science and Humanities, Punjab Agricultural University, Ludhiana, 141004, Punjab, India.

b Indian Institute of Maize Research, Ludhiana, 141004, Punjab, India

* Corresponding author: Principal Scientist, Indian Institute of Maize Research, Ludhiana, 141004, Punjab, India.

e.mail- chaudharydp@gmail.com Phone: 8728900427

**Table S1: Kernel opaqueness, total protein and zein content (% endosperm protein) of Normal, *Opaque-2* and QPM genotype at different stages of kernel development.**

| **Genotype**  **Normal** | **Varieties** | **Opaqueness (%)** | **Prolamin** | | | **Prolamin-like** | | |  | **Residue protein** |  |
| --- | --- | --- | --- | --- | --- | --- | --- | --- | --- | --- | --- |
|  |  |  | **15DAP** | **30DAP** | **45DAP** | **15DAP** | **30DAP** | **45DAP** | **15DAP** | **30DAP** | **45DAP** |
|  | **CML479** | **0** | **28.15 ± 0.02^Ck^** | **34.15 ± 0.03^Bk^** | **41.22± 0.03^Ak^** | **7.14 ± 0.02^Cp^** | **8.66 ± 0.02^Bp^** | **12.94 ±0.04^Ap^** | **17.93±0.03^Cd^** | **13.62±0.09 ^Bd^** | **4.68±0.03 ^Av^** |
|  | **CML334** | **0** | **29.24 ± 0.02^Cg^** | **35.43 ± 0.02^Bg^** | **41.15 ± 0.03^Ag^** | **6.64 ± 0.02^Cu^** | **8.23± 0.01^Bu^** | **11.84± 0.03^Au^** | **20.44±0.06^Cr^** | **16.01±0.03 ^Bp^** | **5.27±0.01 ^At^** |
|  | **CML266** | **0** | **32.27 ± 0.01^Ca^** | **40.11 ± .005^Ba^** | **42.22 ± 0.03^Aa^** | **10.27± 0.01^Cd^** | **12.23± .04^Bd^** | **12.44± 0.01^Ad^** | **19.45±0.05^Cm^** | **12.56±0.03 ^Bu^** | **10.5±0.03 ^As^** |
|  | **CML172** | **0** | **30.65 ± 0.03^Ce^** | **37.44 ± 0.03^Be^** | **40.26 ± 0.03^Ae^** | **8.85 ± 0.02^Ci^** | **10.26± 0.02^Bi^** | **12.64± 0.02^Ai^** | **19.23±0.07^Ce^** | **14.63±0.05 ^Bm^** | **8.87±0.02 ^Aq^** |
|  | **CML169** | **0** | **30.23 ± 0.02^Cd^** | **36.65 ± 0.03^Bd^** | **43.15 ± 0.03^Ad^** | **7.37 ± 0.01^Cm^** | **9.94 ± .04^Bm^** | **12.65± .03^Am^** | **17.04±0.09^Ch^** | **11.74±0.01 ^Bi^** | **2.65±0.02 ^Ao^** |
|  | **CML163** | **0** | **28.25 ± 0.03^Cb^** | **40.44 ± 0.04^Bb^** | **44.07 ± 0.01^Ab^** | **7.46 ± 0.02^Cj^** | **10.23± 0.01^Bj^** | **13.16± 0.03^Aj^** | **23.1±0.07^Cl^** | **12.05±0.02 ^Bn^** | **3.41±0.04 ^Ao^** |
|  | **CML117** | **0** | **27.25 ± .028^Cc^** | **38.85 ± 0.03^Bc^** | **45.22 ± 0.01^Ac^** | **11.15± 0.03^Cb^** | **12.23± .03^Bb^** | **13.96± 0.02^Ab^** | **23.41±0.1^Ck^** | **11.56±0.06 ^Br^** | **3.48±0.09 ^Av^** |
|  | **CML114** | **0** | **27.25 ± .036^Cm^** | **33.45 ± 0.03^Bm^** | **41.75 ± 0.02^Am^** | **7.24 ± 0.02^Cr^** | **8.84 ± 0.03^Br^** | **11.94± 0.02^Ar^** | **19.71±0.08^Cs^** | **14.29±0.08 ^Bj^** | **5.53±0.03 ^An^** |
|  | **CML44** | **0** | **28.24 ± 0.03^Ci^** | **34.46 ± 0.02^Bi^** | **42.27 ± 0.01^Ai^** | **7.26 ± 0.02^Cn^** | **9.92 ± 0.01^Bn^** | **11.94± 0.02^An^** | **19.6±0.06^Ct^** | **14.73±0.03 ^Bs^** | **4.3±0.09 ^Ar^** |
|  | **LM11 1275** | **0** | **28.83 ± 0.03^Ch^** | **34.44 ± 0.02^Bh^** | **42.26 ± 0.02^Ah^** | **7.46 ± 0.02^Cv^** | **8.04 ± 0.02^Bv^** | **10.25± 0.03^Av^** | **19.24±0.03^Ci^** | **15.42±0.08 ^Bv^** | **7.22±0.04 ^Ab^** |
|  | **LM 12** | **0** | **26.26 ± 0.02^Cn^** | **33.44 ± 0.02^Bn^** | **41.24 ± 0.02^An^** | **7.26 ± 0.02^Cv^** | **8.27 ± 0.01^Bv^** | **10.26± 0.02^Av^** | **23.77±0.04^Cp^** | **16.29±0.06 ^Bt^** | **9.41±0.06 ^Aj^** |
|  | **CM 145** | **0** | **27.45 ± 0.036^Cf^** | **36.63 ± 0.02^Bf^** | **42.24 ± 0.03^Af^** | **7.55 ± 0.02^Ct^** | **8.94 ± 0.02^Bt^** | **10.46± 0.02^At^** | **22.26±0.06^Cn^** | **13.75±0.01 ^Bq^** | **7.55±0.04 ^Am^** |
|  | **CM 212** | **0** | **26.64 ± 0.02^Cl^** | **34.45 ± 0.03^Bl^** | **41.96 ± 0.03^Al^** | **7.46 ± 0.02^Cs^** | **9.04 ± 0.02^Bs^** | **10.94± 0.04^As^** | **24.62±0.02^Cj^** | **15.24±0.02 ^Bo^** | **4.13±0.07 ^Ai^** |
|  | **HKI 323(N)** | **0** | **26.25 ± 0.03^Co^** | **32.23 ± 0.03^Bo^** | **41.05 ± 0.03^Ao^** | **8.17 ± 0.01^Cop^** | **9.05 ± 0.01^Bop^** | **11.64± 0.02^Aop^** | **18±0.05^Cq^** | **14.67±0.01 ^Bop^** | **8.57±0.07 ^Aod^** |
|  | **HKI 1105(N)** | **0** | **24.44 ± 0.01^Cp^** | **32.23 ± 0.02^Bp^** | **40.17 ± 0.01^Ap^** | **7.24 ± 0.02^Cq^** | **9.93 ± 0.02^Bq^** | **11.14± 0.01^Aq^** | **21.06±0.04^Co^** | **11.82±0.03 ^Bb^** | **3.58±0.03 ^Au^** |
|  | **HKI 1128(N)** | **0** | **27.74 ± 0.04^Cj^** | **34.44 ± 0.03^Bj^** | **41.94 ± 0.03^Aj^** | **8.04 ± 0.04^Co^** | **8.94 ± 0.01^Bo^** | **11.94 ±0.02^Ao^** | **20.43±0.02^Cv^** | **14.6±0.04 ^Ba^** | **2.48±0.04 ^Ap^** |
| **Mean** |  |  | **28.07±0.9** | **35.54±0.5** | **42.01±0.3** | **7.91±0.9** | **9.54±0.6** | **11.88±0.6** | **20.57±0.03** | **13.94±0.02** | **5.8±0.02** |
| ***Opaque-2*** | **CML269** | **100** | **6.26 ± 0.02^Cv^** | **7.24 ± 0.02^Bv^** | **8.62 ± 0.01^Av^** | **12.65 ± 0.02^Cg^** | **11.16± 0.02^Bg^** | **10.27 ± 0.01^Ag^** | **14.72±0.02 ^Cr^** | **7.96±0.06 ^Bf^** | **5.34±0.05 ^Ah^** |
|  | **DQL 1001** | **100** | **5.25 ± 0.02^Cr^** | **10.22 ± 0.03^Br^** | **8.65 ± 0.02^Ar^** | **12.75 ± 0.02^Cc^** | **11.95± 0.03^Bc^** | **10.85 ± 0.03^Ac^** | **12.99±0.04 ^Cg^** | **8.25±0.05 ^Bh^** | **4.77±0.07 ^Al^** |
|  | **DQL 1005** | **75** | **5.25 ± 0.02^Cu^** | **7.12 ± 0.01^Bu^** | **10.26 ± 0.03^Au^** | **10.34 ± 0.03^Cf^** | **11.25± 0.03^Bf^** | **12.67 ± 0.01^Af^** | **20.01±0.01 ^Cc^** | **6.49±0.05 ^Bh^** | **4.23±0.04 ^Ar^** |
|  | **DQL 1020** | **75** | **4.45 ± 0.03^Cw^** | **6.85 ± 0.03^Bw^** | **10.03 ± 0.02^Aw^** | **10.13 ± 0.01^Ch^** | **10.98± 0.005^Bh^** | **12.24 ± 0.01^Ah^** | **20.67±0.09 ^Cr^** | **9.39±0.09 ^Bop^** | **2.95±0.07 ^Af^** |
|  | **VQL 1** | **50** | **4.47 ± 0.02^Cs^** | **9.14 ± 0.02^Bs^** | **10.06 ± 0.03^As^** | **6.65 ± 0.03^Cl^** | **10.66 ± 0.02^Bl^** | **12.95 ± 0.03^Al^** | **20.81±0.05 ^Cf^** | **7.69±0.06 ^Bc^** | **3.25±0.03 ^Ac^** |
|  | **HKI 323** | **100** | **3.96 ± 0.04^Ct^** | **8.84 ± 0.02^Bt^** | **10.64 ± 0.03^At^** | **6.63 ± 0.005^Cr^** | **9.95 ± 0.02^Br^** | **11.43 ± 0.02^Ar^** | **24.46±0.04 ^Cl^** | **7.09±0.03 ^Bg^** | **5.11±0.03 ^Ag^** |
|  | **HKI 1128** | **100** | **4.45 ± 0.02^Cs^** | **9.25 ± 0.03^Bs^** | **10.05 ± 0.03^As^** | **6.63 ± 0.005^Cop^** | **10.26 ± 0.02^Bop^** | **11.96 ± 0.03^Aop^** | **19.09±0.04 ^Cop^** | **6.72±0.05 ^Br^** | **3.23±0.08 ^Aop^** |
| **Mean** |  |  | **4.88±0.77** | **8.38±0.29** | **9.73±0.77** | **9.4±0.77** | **10.87±0.68** | **11.76±0.97** | **18.98±0.04** | **8±0.07** | **4.18±0.06** |
| **QPM** | **DQL 1019** | **25** | **5.17 ± 0.01^Cy^** | **6.24 ± 0.02^By^** | **9.24 ± 0.02^Ay^** | **9.15 ± 0.02^Ce^** | **12.24 ± 0.02^Be^** | **13.15 ± 0.02^Ae^** | **15.21±0.06 ^Cd^** | **9.94±0.07 ^Baa^** | **1.7±0.05 ^Ax^** |
|  | **LM11 236B** | **25** | **4.43 ± 0.01^Cx^** | **7.95 ± 0.04^Bx^** | **8.64 ± 0.02^Ax^** | **5.95 ± 0.03^Cf^** | **12.18 ± 0.01^Bf^** | **16.15 ± 0.03^Af^** | **25.14±0.04 ^Ck^** | **10.5±0.04 ^Bz^** | **3.04±0.04 ^Az^** |
|  | **LM11 288** | **25** | **4.16 ± 0.03^Caa^** | **7.16 ± 0.03^Baa^** | **8.15 ± 0.02^Aaa^** | **6.65 ± 0.02^Cd^** | **12.25 ± 0.04^Bd^** | **16.06 ± 0.04^Ad^** | **23.870.09 ^Cl^** | **9.05±0.06 ^By^** | **1.53±0.09 ^At^** |
|  | **LM12 205** | **0** | **4.05 ± 0.02^Cz^** | **7.94 ± 0.04^Bz^** | **8.23 ± 0.02^Az^** | **5.95 ± 0.04^Cd^** | **12.92± 0.005^Bd^** | **16.16 ± 0.03^Ad^** | **23.42±0.04 ^Cf^** | **9.85±0.04 ^Bt^** | **5.13±0.04 ^Aq^** |
|  | **LM12 177** | **25** | **5.17 ± 0.01^Cy^** | **7.26 ± 0.03^By^** | **8.19 ± 0.005^Ay^** | **5.93 ± 0.01^Ck^** | **10.14 ± 0.03^Bk^** | **14.43 ± 0.02^Ak^** | **24.21±0.07 ^Cd^** | **9.85±0.05 ^By^** | **6.94±0.04 ^Ay^** |
|  | **VQL 2** | **25** | **4.46 ± 0.02^Ct^** | **9.02 ± 0.01^Bt^** | **9.95 ± 0.02^At^** | **6.65 ± 0.02^Cl^** | **10.15 ± 0.03^Bl^** | **13.34 ± 0.03^Al^** | **18.4±0.08 ^Ca^** | **7.24±0.08 ^Bq^** | **0.93±0.06 ^Aaa^** |
|  | **HKI 1105** | **25** | **6.23 ± 0.02^Cq^** | **9.45 ± 0.02^Bq^** | **10.04 ± 0.01^Aq^** | **9.03 ± 0.02^Ca^** | **13.83 ± 0.02^Ba^** | **18.04 ± 0.02^Aa^** | **9.17±0.06 ^Ce^** | **5.62±0.07 ^Bx^** | **2.13±0.07 ^Ay^** |
| **Mean** |  |  | **4.8±0.78** | **7.86±0.71** | **8.92±0.8** | **7.06±0.4** | **11.96±0.4** | **14.91±0.3** | **19.92±0.03** | **8.86±0.03** | **3.05±0.06** |
| **Overall Mean** |  |  | **17.23 ± 0.95** | **22.75±0.41** | **28.32±0.71** | **8.06±0.8** | **10.42±0.5** | **12.84±0.9** | **19.81±0.8** | **10.27±0.4** | **4.34±1.2** |

**_*Values are mean ± SD of three replicates, values for zein fractions (prolamin, prolamin-like) and residue protein are expressed as protein percentage with respect to total protein content. Values with same letter(s) in a column are not significantly different at P ≤ 0.05 (Tukey’s post-hoc test)._**

**Table S2: Non-zein content of Normal, *Opaque-2* and QPM genotypes at different stages of kernel development.**

| **Genotype**  **Normal** | **Varieties** | **Albumin** | | | **Globulin** | | | **Glutelin-like** | | | **Glutelin** | | |
| --- | --- | --- | --- | --- | --- | --- | --- | --- | --- | --- | --- | --- | --- |
|  |  | **15DAP** | **30DAP** | **45DAP** | **15DAP** | **30DAP** | **45DAP** | **15DAP** | **30DAP** | **45DAP** | **15DAP** | **30DAP** | **45DAP** |
|  | **CML479** | **14.44 ± 0.02^An^** | **9.95 ± 0.01^Bn^** | **5.14 ± 0.03^Cn^** | **9.14 ± 0.02^Ao^** | **7.23 ± 0.01^Bo^** | **6.13 ± 0.037^Co^** | **6.96 ± 0.03^Cl^** | **9.15 ± 0.03^Bl^** | **11.24 ± 0.03^Al^** | **16.24 ± 0.02^Cr^** | **17.24 ± 0.02^Br^** | **18.65 ± 0.02^Ar^** |
|  | **CML334** | **14.23 ± 0.02^Ar^** | **8.83 ± 0.037^Br^** | **5.06 ± 0.037^Cr^** | **8.15 ± 0.015^As^** | **6.44 ± 0.02^Bs^** | **5.84 ± 0.03^Cs^** | **5.85 ± 0.03^Co^** | **8.43 ± 0.01^Bo^** | **12.43 ± 0.03^Ao^** | **15.45 ± 0.02^Cu^** | **16.63 ± 0.02^Bu^** | **18.41 ± 0.02^Au^** |
|  | **CML266** | **15.16 ± 0.02^Al^** | **10.23 ± 0.01^Bl^** | **6.63 ± 0.032^Cl^** | **9.17 ± 0.015^Ao^** | **7.47 ± 0.02^Bo^** | **5.95 ± 0.03^Co^** | **5.24 ± 0.03^Cx^** | **7.15 ± 0.02^Bx^** | **10.14 ± 0.03^Ax^** | **8.44 ± 0.03^Caa^** | **10.25 ± 0.02^Baa^** | **11.12 ± 0.02^Aaa^** |
|  | **CML172** | **14.44 ± 0.03^Am^** | **9.46 ± 0.02^Bm^** | **6.87 ± 0.01^Cm^** | **8.94 ± 0.04^Aq^** | **7.14 ± 0.02^Bq^** | **5.77 ± 0.015^Cq^** | **5.63 ± 0.02^Cs^** | **7.74 ± 0.03^Bs^** | **11.17 ± 0.01^As^** | **12.26 ± 0.02^Cz^** | **13.33 ± 0.02^Bz^** | **14.42 ± 0.02^Az^** |
|  | **CML169** | **13.95 ± 0.035^Ap^** | **8.84 ± 0.02^Bp^** | **5.86 ± 0.037^Cp^** | **8.04 ± 0.03^Ar^** | **6.94 ± 0.04^Br^** | **6.03 ± 0.015^Cr^** | **6.64 ± 0.02^Cp^** | **8.14 ± 0.02^Bp^** | **11.67 ± 0.01^Ap^** | **16.73 ± 0.03^Cq^** | **17.75 ± 0.02^Bq^** | **17.99 ± 0.02^Aq^** |
|  | **CML163** | **12.75 ± 0.026^At^** | **7.95 ± 0.036^Bt^** | **6.04 ± 0.02^Ct^** | **8.16 ± 0.025^At^** | **5.43 ± 0.01^Bt^** | **5.77 ± 0.015^Ct^** | **5.23 ± 0.01^Cv^** | **7.27 ± 0.01^Bv^** | **10.54 ± 0.03^Av^** | **15.05 ± 0.03^Cw^** | **16.63 ± 0.02^Bw^** | **17.01 ± 0.02^Aw^** |
|  | **CML117** | **11.94 ± 0.03^Aw^** | **7.75 ± .026^Bw^** | **5.15 ± .03^Cw^** | **6.76 ± 0.025^Au^** | **5.44 ± 0.03^Bu^** | **5.26 ± 0.02^Cu^** | **5.03 ± 0.02^Cw^** | **7.44 ± 0.02^Bw^** | **10.24 ± 0.02^Aw^** | **14.46 ± 0.02^Cy^** | **16.73 ± 0.03^By^** | **16.69 ± 0.03^Ay^** |
|  | **CML114** | **14.45 ± 0.036^Aq^** | **8.66 ± 0.025^Bq^** | **5.27 ± 0.015^Cq^** | **9.45 ± 0.036^Aj^** | **8.33 ± 0.01^Bj^** | **6.85 ± 0.026^Cj^** | **5.86 ± 0.02^Cr^** | **8.96 ± 0.02^Br^** | **10.24 ± 0.02^Ar^** | **16.04 ± 0.02^Cs^** | **17.47 ± 0.02^Bs^** | **18.42 ± 0.02^As^** |
|  | **CML44** | **13.46 ± 0.02^As^** | **8.35 ± 0.025^Bs^** | **5.95 ± 0.037^Cs^** | **8.93 ± 0.01^Ar^** | **6.44 ± 0.025^Br^** | **5.60 ± 0.01^Cr^** | **6.56 ± 0.02^Cm^** | **8.64 ± 0.02^Bm^** | **11.96 ± 0.03^Am^** | **15.95 ± 0.03^Ct^** | **17.46 ± 0.02^Bt^** | **17.98 ± 0.02^At^** |
|  | **LM11 1275** | **13.24 ± 0.02^As^** | **8.97 ± 0.015^Bs^** | **5.66 ± 0.011^Cs^** | **9.42 ± 0.015^Aj^** | **8.25 ± 0.03^Bj^** | **6.96 ± 0.04^Cj^** | **5.86 ± 0.01^Ct^** | **8.24 ± 0.03^Bt^** | **10.23 ± 0.01^At^** | **15.95 ± 0.02^Cv^** | **16.64 ± 0.02^Bv^** | **17.42 ± 0.02^Av^** |
|  | **LM 12** | **13.26 ± 0.02^Au^** | **7.96 ± 0.025^Bu^** | **5.22 ± 0.011^Cu^** | **8.96 ± 0.025^Al^** | **8.76 ± 0.02^Bl^** | **6.43 ± 0.02^Cl^** | **6.16 ± 0.02^Cr^** | **8.84 ± 0.02^Br^** | **9.95 ± 0.01^Ar^** | **14.33 ± 0.02^Cx^** | **16.44 ± 0.03^Bx^** | **17.49 ± 0.03^Ax^** |
|  | **CM 145** | **12.94 ± 0.02^Au^** | **8.25 ± 0.03^Bu^** | **5.16 ± 0.01^Cu^** | **9.76 ± 0.005^Akl^** | **8.24 ± 0.02^Bkl^** | **6.27 ± 0.015^Ckl^** | **5.07 ± 0.02^Cu^** | **8.42 ± 0.01^Bu^** | **10.54 ± 0.03^Au^** | **14.97 ± 0.01^Cw^** | **15.95± 0 .03^Bw^** | **17.78 ± 0.03^Aw^** |
|  | **CM 212** | **11.14 ± 0.02^Av^** | **8.92 ± 0.015^Bv^** | **6.05 ± 0.03^Cv^** | **9.91 ± 0.01^Ai^** | **8.46 ± 0.02^Bi^** | **6.93 ± 0.03^Ci^** | **5.06 ± 0.01^Cu^** | **7.95 ± 0.03^Bu^** | **11.13 ± 0.01^Au^** | **15.17 ± 0.01^Cv^** | **15.94 ± 0.04^Bv^** | **18.86 ± 0.04^Av^** |
|  | **HKI 323(N)** | **15.55 ± 0.025^Aq^** | **8.46 ± 0.025^Bq^** | **4.31 ± 0.01^Cq^** | **10.16 ± 0.015^Ap^** | **7.45 ± 0.02^Bp^** | **4.53 ± 0.03^Cp^** | **6.02± 0.005^Cq^** | **9.97 ± 0.01^Bq^** | **10.25 ± 0.02^Aq^** | **15.85 ± 0.02^Cp^** | **18.17 ± 0.01^Bp^** | **19.65 ± 0.01^Ap^** |
|  | **HKI 1105(N)** | **14.46 ± 0.02^Ao^** | **8.77 ± 0.01^Bo^** | **6.06 ± 0.02^Co^** | **9.61 ± 0.02^Ag^** | **8.95 ± 0.03^Bg^** | **7.16 ± 0.02^Cg^** | **5.45 ± 0.01^Cn^** | **9.84 ± 0.02^Bn^** | **11.65 ± 0.02^An^** | **17.74 ± 0.02^Cn^** | **18.46 ± 0.02^Bn^** | **20.24 ± 0.02^An^** |
|  | **HKI 1128(N)** | **12.44 ± 0.02^At^** | **7.91 ± 0.005^Bt^** | **5.84 ± 0.02^Ct^** | **9.12 ± 0.03^Akl^** | **8.24 ± 0.03^Bkl^** | **6.93 ± 0.01^Ckl^** | **5.59 ± 0.01^Cr^** | **8.43 ± 0.01^Br^** | **10.95 ± 0.03^Ar^** | **16.64 ± 0.02^Co^** | **17.44 ± 0.02^Bo^** | **19.92 ± 0.02^Ao^** |
| **Mean** |  | **13.65 ± 0.1** | **8.71 ± 0.7** | **5.64 ± 0.6** | **8.96 ± 0.8** | **7.45 ± 0.1** | **6.14 ± 0.7** | **5.76 ± 0.5** | **8.41 ± 0.8** | **10.9 ± 0.7** | **15.08 ± 0.1** | **16.41 ± 0.2** | **17.63 ± 0.2** |
| ***Opaque-2*** | **CML269** | **14.16 ± 0.02^Ah^** | **12.13 ± 0.01^Bh^** | **10.24 ± 0.02^Ch^** | **10.13 ± 0.01^Ab^** | **9.66 ± 0.01^Bb^** | **8.26 ± 0.02^Cb^** | **16.63 ± 0.02^Cj^** | **18.24 ± 0.03^Bj^** | **20.62 ± 0.01^Aj^** | **25.45 ± 0.02^Ca^** | **33.61± 0.005^Ba^** | **36.65 ±0.03^Aa^** |
|  | **DQL 1001** | **13.66 ± 0.02^Ah^** | **11.85 ± 0.02^Bh^** | **11.16 ± 0.02^Ch^** | **10.24 ± 0.02^Aa^** | **9.44 ± 0.02^Ba^** | **8.96 ± 0.02^Ca^** | **17.17 ± 0.01^Ci^** | **17.85 ± 0.03^Bi^** | **20.66 ± 0.02^Ai^** | **27 .94 ± 0.03^Cd^** | **30.44± 0.02^Bd^** | **34.95± 0.03^Ad^** |
|  | **DQL 1005** | **15.64 ± 0.03^Ad^** | **13.05 ± 0.03^Bd^** | **12.04 ± 0.02^Cd^** | **9.94 ± 0.03^Ac^** | **9.64 ± 0.02^Bc^** | **8.21 ± 0.005^Cc^** | **17.24 ± 0.03^Ch^** | **19.05 ± 0.03^Bh^** | **20.34 ± 0.01^Ah^** | **21.58 ± 0.02^Ch^** | **33.4± 0.005^Bh^** | **32.25± 0.03^Ah^** |
|  | **DQL 1020** | **15.63 ± 0.02^Ac^** | **13.04 ± 0.04^Bc^** | **12.24 ± 0.02^Cc^** | **9.14 ± 0.03^Af^** | **9.17 ± 0.03^Bf^** | **8.07 ± 0.01^Cf^** | **18.82 ± 0.01^Cd^** | **20.24 ± 0.02^Bd^** | **22.23 ± 0.04^Ad^** | **21.16 ± 0.03^Cm^** | **30.33 ± 0.02^Bm^** | **32.24± 0.02^Am^** |
|  | **VQL 1** | **15.83 ± 0.03^Af^** | **10.96 ± 0.02^Bf^** | **11.06 ± 0.03^Cf^** | **9.63 ± 0.02^Ad^** | **9.74 ± 0.02^Bd^** | **8.17 ± 0.01^Cd^** | **17.46 ± 0.02^Cc^** | **21.18 ± 0.01^Bc^** | **23.33 ± 0.03^Ac^** | **25.15 ± 0.03^Ci^** | **30.63 ± 0.02^Bi^** | **31.18± 0.01^Ai^** |
|  | **HKI 323** | **14.96 ± 0.02^Ag^** | **11.15 ± 0.03^Bg^** | **10.93 ± 0.03^Cg^** | **9.05 ± 0.03^Ae^** | **9.66 ± 0.01^Be^** | **8.04 ± 0.02^Ce^** | **16.67 ± 0.02^Cb^** | **23.36 ± 0.03^Bb^** | **23.51 ± 0.02^Ab^** | **24.27 ± 0.02^Cl^** | **29.95 ± 0.02^Bl^** | **30.34± 0.02^Al^** |
|  | **HKI 1128** | **19.86 ± 0.03^Ab^** | **12.64 ± 0.02^Bb^** | **12.93 ± 0.02^Cb^** | **8.85 ± 0.02^Ac^** | **9.93 ± 0.03^Bc^** | **9.04 ± 0.03^Cc^** | **16.66 ± 0.03^Ce^** | **20.25 ± 0.03^Be^** | **23.34 ± 0.03^Ae^** | **24.46 ± 0.03^Ck^** | **30.95 ± 0.04^Bk^** | **29.45± 0.03^Ak^** |
| **Mean** |  | **15.68 ± 0.9** | **12.09 ± 0.8** | **11.52 ± 0.9** | **9.56 ± 0.5** | **9.3 ± 0.23** | **8.39 ± 0.42** | **17.22 ± 0.7** | **20.03 ± 0.8** | **22 ± 0.4** | **24.28 ± 0.3** | **31.33 ± 0.5** | **32.42 ± 0.5** |
| **QPM** | **DQL 1019** | **20.26 ± 0.02^Ab^** | **12.24 ± 0.03^Bb^** | **13.07 ± 0.04^Cb^** | **8.54 ± 0.04^Af^** | **9.15 ± 0.02^Bf^** | **8.64 ± 0.02^Cf^** | **18.23 ± 0.01^Cf^** | **20.13 ± 0.04^Bf^** | **21.07 ± 0.01^Af^** | **23.44 ± 0.01^Cj^** | **30.06 ± 0.02^Bj^** | **33.13± 0.02^Aj^** |
|  | **LM11 236B** | **13.16 ± 0.02^Ak^** | **10.22 ± 0.01^Bk^** | **9.95 ± 0.04^Ck^** | **8.32 ± 0.01^Ak^** | **8.04 ± 0.04^Bk^** | **7.96 ± 0.03^Ck^** | **17.05 ± 0.04^Ck^** | **18.05 ± 0.03^Bk^** | **19.93 ± 0.01^Ak^** | **25.95 ± 0.04^Cd^** | **33.06 ± 0.04^Bd^** | **34.42± 0.01^Ad^** |
|  | **LM11 288** | **14.05 ± 0.04^Aj^** | **10.23 ± 0.02^Bj^** | **10.01 ± 0.06^Ch^** | **8.98 ±0.08 ^Ah^** | **8.61 ±0.06^Bm^** | **7.96 ±0.08^Ce^** | **16.93 ±0.06^Cf^** | **20.26 ± 0.02^Be^** | **21.86 ± 0.02^Ag^** | **26.17 ± 0.01^Cc^** | **33.04 ± 0.03^Bc^** | **34.43± 0.03^Ac^** |
|  | **LM12 205** | **13.04 ± 0.03^Ak^** | **10.26 ± 0.02^Bk^** | **9.95 ± 0.02^Ck^** | **8.64 ± 0.02^Am^** | **8.03 ± 0.01^Bm^** | **7.22 ± 0.005^Cm^** | **16.65 ± 0.03^Ci^** | **18.84 ± 0.02^Bi^** | **20.26 ± 0.03^Ai^** | **28.25 ± 0.02^Cb^** | **32.96 ± 0.03^Bb^** | **33.05± 0.03^Ab^** |
|  | **LM12 177** | **14.45 ± 0.03^Ai^** | **11.15 ± 0.03^Bi^** | **9.15 ± 0.03^Ci^** | **8.95 ± 0.03^Agh^** | **8.72 ± 0.02^Bgh^** | **7.94 ± 0.03^Cgh^** | **16.95 ± 0.03^Cg^** | **20.95 ± 0.01^Bg^** | **21.17 ± 0.01^Ag^** | **24.34 ± 0.02^Cg^** | **31.93 ± 0.02^Bg^** | **32.18 ±0.01^Ag^** |
|  | **VQL 2** | **16.06 ± 0.03^Ae^** | **11.03 ±0.005^Be^** | **11.16 ± 0.01^Ce^** | **9.04 ± 0.03^Ac^** | **9.93 ± 0.02^Bc^** | **8.83 ± 0.01^Cc^** | **17.95 ± 0.03^Ca^** | **22.07 ± 0.02^Ba^** | **24.66 ± 0.02^Aa^** | **27.44 ± 0.02^Cf^** | **30.56 ± 0.02^Bf^** | **31.13± 0.04^Af^** |
|  | **HKI 1105** | **21.27 ± 0.01^Aa^** | **13.07 ±0.015^Ba^** | **11.25 ± 0.03^Ca^** | **9.15 ± 0.01^An^** | **7.46 ± 0.03^Bn^** | **5.04 ± 0.03^Cn^** | **17.74 ± 0.04^Ce^** | **20.24 ± 0.03^Be^** | **22.07 ± 0.01^Ae^** | **27.41 ±0.005^Ce^** | **30.33 ± 0.01^Be^** | **31.99± 0.04^Ae^** |
| **Mean** |  | **16.03±0.3** | **11.18±0.71** | **11.07±0.8** | **8.8±0.29** | **8.55±0.83** | **7.79±0.93** | **17.36±0.6** | **20.07±0.3** | **21.57±0.5** | **26.15±0.7** | **31.71±0.3** | **32.9±0.2** |
| **Overall Mean** |  | **14.67±0.2** | **10.07±0.7** | **8.28±0.76** | **9.07±0.7** | **8.21±0.2** | **7.08±0.2** | **11.13±0.8** | **13.83±0.5** | **15.99±0.6** | **21.14±0.3** | **23.75±0.92** | **24.68±0.9** |

**_Values are mean ± SD of three replicates, values for non-zein fractions (albumin, globulin, glutelin-like, glutein) are expressed as protein percentage with respect to total protein content. Values with same letter(s) in a column are not significantly different at P ≤ 0.05 (Tukey’s post-hoc test)._**
